# Proteome Characterization of Leaves in Common Bean

**DOI:** 10.3390/proteomes3030236

**Published:** 2015-08-18

**Authors:** Faith M. Robison, Adam L. Heuberger, Mark A. Brick, Jessica E. Prenni

**Affiliations:** 1 Proteomics and Metabolomics Facility, Colorado State University, Fort Collins, CO 80523, USA; E-Mail: fmr33@cornell.edu; 2 Department of Soil and Crop Sciences, Colorado State University, Fort Collins, CO 80523, USA; E-Mail: Mark.Brick@ColoState.EDU; 3 Department of Horticulture and Landscape Architecture, Colorado State University, Fort Collins, CO 80523, USA; E-Mail: Adam.Heuberger@colostate.edu; 4 Department of Biochemistry, Colorado State University, Fort Collins, CO 80523, USA

**Keywords:** shotgun proteomics, LC-MS, *Phaseolus vulgaris*, plant defense, pathogen resistance, beans, leaves

## Abstract

Dry edible bean (*Phaseolus vulgaris* L.) is a globally relevant food crop. The bean genome was recently sequenced and annotated allowing for proteomics investigations aimed at characterization of leaf phenotypes important to agriculture. The objective of this study was to utilize a shotgun proteomics approach to characterize the leaf proteome and to identify protein abundance differences between two bean lines with known variation in their physiological resistance to biotic stresses. Overall, 640 proteins were confidently identified. Among these are proteins known to be involved in a variety of molecular functions including oxidoreductase activity, binding peroxidase activity, and hydrolase activity. Twenty nine proteins were found to significantly vary in abundance (*p*-value < 0.05) between the two bean lines, including proteins associated with biotic stress. To our knowledge, this work represents the first large scale shotgun proteomic analysis of beans and our results lay the groundwork for future studies designed to investigate the molecular mechanisms involved in pathogen resistance.

## 1. Introduction

Dry edible bean (*Phaseolus vulgaris* L., “bean”) is an important food crop that is grown and consumed worldwide [1]. Molecular techniques are critical to bean research to facilitate the development of high-yielding, disease resistant bean lines adapted to a wide range of environments. Current efforts in bean breeding are focused on the identification of genetic markers for important plant phenotypes in agriculture, including yield and resistance to abiotic and biotic stresses [2]. However, the functional effect of genetic markers on a plant phenotype is often not well understood and therefore additional molecular techniques focused on the analysis of RNA, proteins or metabolites, are necessary to characterize the genetic basis of plant phenotypes [3,4].

To date, the majority of plant proteomic studies have utilized 2-dimensional (2-D) gel electrophoresis for protein separation and quantitation, followed by either matrix-assisted laser desorption/ionization-mass spectrometry coupled to time of flight (MALDI-TOF) or liquid chromatography coupled to tandem mass spectrometry (LC-MS/MS) [5,6,7,8,9,10,11,12]. For example, Katam *et al.* conducted studies on peanut leaves utilizing 2-D gels in conjunction with MALDI-TOF in which they were able to visualize 300 protein spots and confidently identify 174 proteins [13]. Salavati *et al.* used a similar approach and reported visualization of 483 protein spots for the common bean proteome. Based on quantitative differences observed in the 2-D gels, 32 identified proteins were involved in the early stage of symbiosis between bean and the nitrogen fixing bacterium *Rhizobium et al*. [14]. Zadraznik *et al.* used an approach combining 2-D gels and LC-MS/MS to characterize proteome changes resulting from drought stress in bean leaves [6]. A recent study utilized a shotgun proteomics approach in which they report the detection and identification of 2600 proteins from cassava root, 300 of which showed significant changes during postharvest physiological deterioration [15]. 

In the study presented here, the bean leaf proteome was characterized using a shotgun LC-MS/MS approach and a comparison was performed between two bean lines that are genetically similar but contain differences in susceptibility to leaf pathogens. To our knowledge, this study represents the first comprehensive proteome characterization of bean leaves and illustrates the utility of this approach for investigations of disease response mechanisms in plants.

## 2. Experimental Section

### 2.1. Plant Material and Growth Conditions

Bean lines are derived from two major centers of domestication: Mesoamerican (small seeded types, e.g., navy, pinto) or Andean (large-seed types, e.g., kidney, alubia). Two common bean lines of Andean origin, A195 and Sacramento, were included in the analysis. A195 is large-seeded dry bean line developed at the International center for Tropical Agriculture in Colombia [16]. Sacramento is a commercial large-seeded light red kidney bean cultivar released by Sacramento Valley Milling in 1975 [17]. The two lines differ in their physiological response to white mold (caused by *Sclerotinia sclerotiorum*, angular leaf spot (caused by *Phaeoisariopsis griseola (Sacc.) Ferraris*), and bean common mosaic virus (BCMV) a non-persistent, stylet-borne, aphid-transmitted virus, with A195 being resistant and Sacramento being susceptible to all three diseases [16,18,19].

Beans (*n* = 3 for each line) were planted in 4.5 L pots with high porosity potting media that was treated with Root Shield (*Trichoderma harzianum*, strain T-22). A pre-plant control for fungus gnats (*Bradysia* species) and 10 g osmocote 19-5-8 time release fertilizer with micronutrients was incorporated pre-planting. After planting, the pots were watered to field capacity (water draining out the bottom of the pots). Plants were grown in a greenhouse with a photoperiod of a 16/8 h light/dark and were watered once a day using a timed irrigation system. The greenhouse heating set-points were 20 °C during the day and 17 °C at night, and the cooling set points were 26 °C during the day and 24 °C at night. The relative humidity was maintained at 55% during the day and 75% at night.

### 2.2. Protein Extraction

Healthy leaves were detached at the petiole and tissue was acquired using a 1 cm diameter leaf hole punch (approximately 12 mg dry weight per punch). A total of five punches per leaf were acquired, transferred to a 1.5 mL microcentrifuge tube and flash frozen in liquid nitrogen. Frozen tissue was ground to a fine powder using 1 cm diameter steal beads with a TissueLyser II (Qiagen, Germantown, MD, USA). Protein was extracted by the addition of 1 mL of methanol:water (80:20, *v*:*v*), vortexing for 2 h at room temperature, and incubation at −80 °C for 20 min. The sample was centrifuged at 16,000× *g* for 20 min at 4 °C, the supernatant was removed and the pellet was dried using a Savant speedvac (Thermo Scientific, Waltham, MA, USA). Three hundred microliters of 8 M urea was added to the pellet to solubilize protein and the sample was vortexed at room temperature for 15 min. Samples were centrifuged again at 10,000× *g* for 10 min and the supernatant containing the proteins was used for further analysis.

### 2.3. Protein Digestion

Total protein concentration was determined using a Bradford assay (Thermo Scientific) following manufacture protocols. From each sample, 30 μg of total protein was aliquoted and then precipitated by adding four volumes of −20 °C acetone, followed by a short vortex and incubation at −80 °C for 20 min. Samples were centrifuges at 10,000× *g* for 10 min at 4 °C. The protein pellet was washed with 300 μL of (−20 °C) acetone and centrifuged at 10,000× *g* for 5 min at 4 °C. The supernatant was discarded and the pellet was air dried in a dust free environment.

The dry protein pellet was solubilized using 15 μL of 8 M urea and bath sonicated for 5 min. Then, 20 μL of 0.2% (by volume) ProteaseMAX™ Surfactant (Promega, Madison, WI, USA) was added and mixed by shaking on vortexer for 5–10 min. Afterwards, 58.5 μL of 50 mM ammonium bicarbonate was added to achieve a final volume of 93.5 μL. One microliter of 0.5 M DTT (1,4-dithiothreitol) was added and incubated at 50 °C for 20 min. Then, 2.7 μL of 0.55 M IAA (iodoacetic acid) was added and incubated at room temperature in the dark for 15 min. One microliter of 1% (by volume) ProteaseMAX and 18 μL of 0.1 μg/μL trypsin (Promega) were added. Samples were incubated at 37 °C for 3 h. After the digestion was complete, samples were briefly centrifuged and 27 μL of 2.5% (by volume) TFA (trifluoroacetic acid) was added to stop enzymatic digestion. Samples were then mixed, and incubated at room temperature for 5 min. The peptides were desalted using Pierce C18 spin columns (per manufactures protocol) and dried in a speedvac. Samples were then prepared for mass spectrometry by resuspension in 5% acetonitrile (ACN)/1% formic acid (by volume) to a concentration of 1.0 μg/μL.

### 2.4. Mass Spectrometry

Peptides were further purified and concentrated using an on-line enrichment column (5 μm, 100 μm ID × 2cm C18 column, Thermo Scientific, Cat#: 89870). One microliter of sample was injected in 100% buffer A (0.1% formic acid, by volume) at a flow rate of 20 μL/min (20 μL injection loop) with enrichment trapping at a flow rate of 4 μL/min. Subsequent chromatographic separation was performed on a reverse phase nanospray column (EASYnano-LC, 3 μm, 75 μm ID × 100 mm C18 column, Thermo Scientific) using a 90 min linear gradient from 10%–30% buffer B (100% ACN, 0.1% formic acid by volume) at a flow rate of 400 nL/min. Peptides were eluted directly into the mass spectrometer (Orbitrap Velos, Thermo Scientific) equipped with a Nanospray Flex ion source (Thermo Scientific) and spectra were collected over a *m*/*z* range of 400–2000, positive mode ionization, using a dynamic exclusion limit of 2 MS/MS spectra of a given *m*/*z* value for 30 s (exclusion duration of 90 s). The instrument was operated in FT mode for MS detection (resolution of 60,000) and ion trap mode for MS/MS detection with a normalized collision energy set to 35%. Compound lists of the resulting spectra were generated using Xcalibur software (Thermo Scientific, version 2.2) with a S/N threshold of 1.5 and 1 scan/group. 

### 2.5. Data Analysis

MS/MS spectra were searched against the *Phaseolus vulgaris* protein sequence database obtained from Phytozome.net and concatenated to a reverse database (63,276 sequence entries, version 16 May 2013) using the Mascot database search engine (version 2.3, Matrix Science, Boston, MA, USA). The following search parameters were included: monoisotopic mass, parent ion mass tolerance of 20 ppm, fragment ion mass tolerance of 0.8 Da, fully tryptic peptides with one missed cleavage, variable modification of oxidation of methionine and fixed modification of carbamidomethylation of cysteine.

Search results for each sample were imported and combined using probabilistic protein identification algorithms [20] implemented in Scaffold software [21] (version 4, Proteome Software, Portland, OR, USA ). A peptide threshold of 99% and protein probability threshold of 90% were applied and a minimum of two unique peptides was required. A peptide false discovery rate was calculated by Scaffold based on hits to the reverse database [22]. Proteins species that contained similar peptides and could not be differentiated based on MS/MS analysis alone were grouped to satisfy the principles of parsimony. 

Instrument functionality and stability was monitored using the MassQC software (Proteome Software). This software uses a set of quantitative metrics developed by the National Institute of Science and Technology (NIST) in collaboration with the National Cancer Institute’s Clinical Proteomic Technologies for Cancer (CPTC) that monitor technical variability in mass spectrometry-based proteomics instrumentation [23]. Quality control samples containing a mixture of 6 trypsin digested bovine proteins were injected at least once every 24 h throughout the analysis, and the data from this run was analyzed using the MassQC software. Values for all metrics were within normal limits throughout the duration of the experiment indicating instrument stability and data robustness.

Relative quantitation was determined using both spectral counting (SpC) and average MS/MS total ion current (MS^2^ TIC). [24,25]. Data was normalized using the default method in the Scaffold software. A student’s t-test was applied to determine protein species that were significantly different in abundance between groups (*p*-value < 0.05). The resulting list of significantly different proteins was further filtered by the following criteria: proteins must be present in a minimum of 2 out of 3 biological replicates for a given group and the total normalized spectral counts for a given group must be >10. Pseudo values were added (+1 for SpC and +1000 for MS^2^ TIC) prior to fold change calculations to eliminate zero values.

Gene ontology (GO) term enrichment analysis was performed in Pathway Studio Plant Web (Elsevier, Atlanta, GA, USA) and was based on *Arabidopsis thaliana* GO libraries (https://www.arabidopsis.org). Gene Set Enrichment Analysis (GSEA) [26] was conducted in Pathway Studio Plant Web using the Mann-Whitney-U Test, and significantly enriched pathways were determined using a threshold of *p* < 0.05.

## 3. Results and Discussion

**Figure 1:**
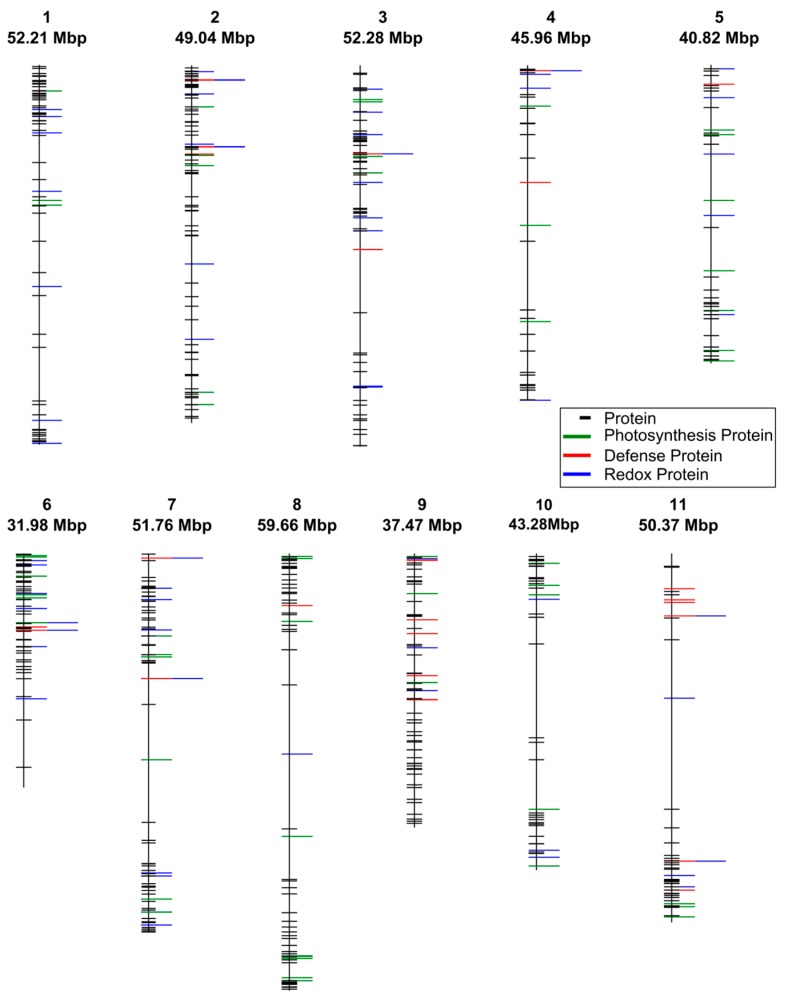
Genomic location of detected protein species using shotgun LC-MS/MS. Genomic locations were determined by aligning detected protein annotations with gene locations. Colors indicate proteins involved in photosynthesis (**green**), plant defense (**red**), or redox (**blue**).

In total, 640 protein species were confidently identified (Supplementary Table S1). Of these, 184 protein species were detected in all six leaf samples. The 640 identified protein species were mapped to genetic loci within the bean genome to evaluate the potential for shotgun proteomics to facilitate investigations into quantitative genetics, specifically protein quantitative trait loci (pQTL). Chromosome location and length was determined by aligning the detected protein annotations with transcript annotations and corresponding genomic loci provided by Phytozome.net [27] (Supplementary Table S2). Gene sequences related to the detected protein species spanned across all 11 of the *Phaseolus vulgaris* chromosomes (Figure 1). This analysis revealed a wide genomic distribution of the 640 detected leaf protein species with chromosome 9 being most saturated. Further, molecular processes related to photosynthesis, response to biotic stress and redox status were also widely distributed across the genome. These results support shotgun proteomics of plant leaves as a valuable tool to investigate genetic resistance to biotic stress.

The data was subsequently interrogated for co-localization of genes that encode proteins associated with a physiological or molecular response to biotic stress. For example, two basic chitinases (Phvul.009G116600.1 and Phvul.009G116700.1) co-localized on chromosome 9 with starting positions at 17,449,427 bp and 17,485,614 bp, respectively. Two of the three detected osmotin 34 protein genes, (Phvul.002G286500.1 and Phvul.002G286600.1) are located on chromosome 2 with starting positions at 45,043,859 bp and 45,053,480, respectively. Conversely, some of the detected protein species contained genes that do not co-localize, such as polygalacturonase inhibiting protein 1s which is found on chromosome 2 and chromosome 9 with starting positions at 36,117,844 bp on chromosome 2 and 26,512,436 bp on chromosome 9, respectively. This is consistent with several protein homologues that were detected, such as photosystem II subunit O-2 (chromosome 2 and 3) and photosystem I subunit E-2 (chromosome 7 and 8). Overall, the shotgun proteomics approach resulted in the detection of leaf protein species representative of the bean genome. Taken together, these results illustrate the potential to integrate shotgun proteomic data with traditional quantitative genetics in the context of plant based molecular investigations.

### 3.1. Characterization of Bean Proteins

**Figure 2:**
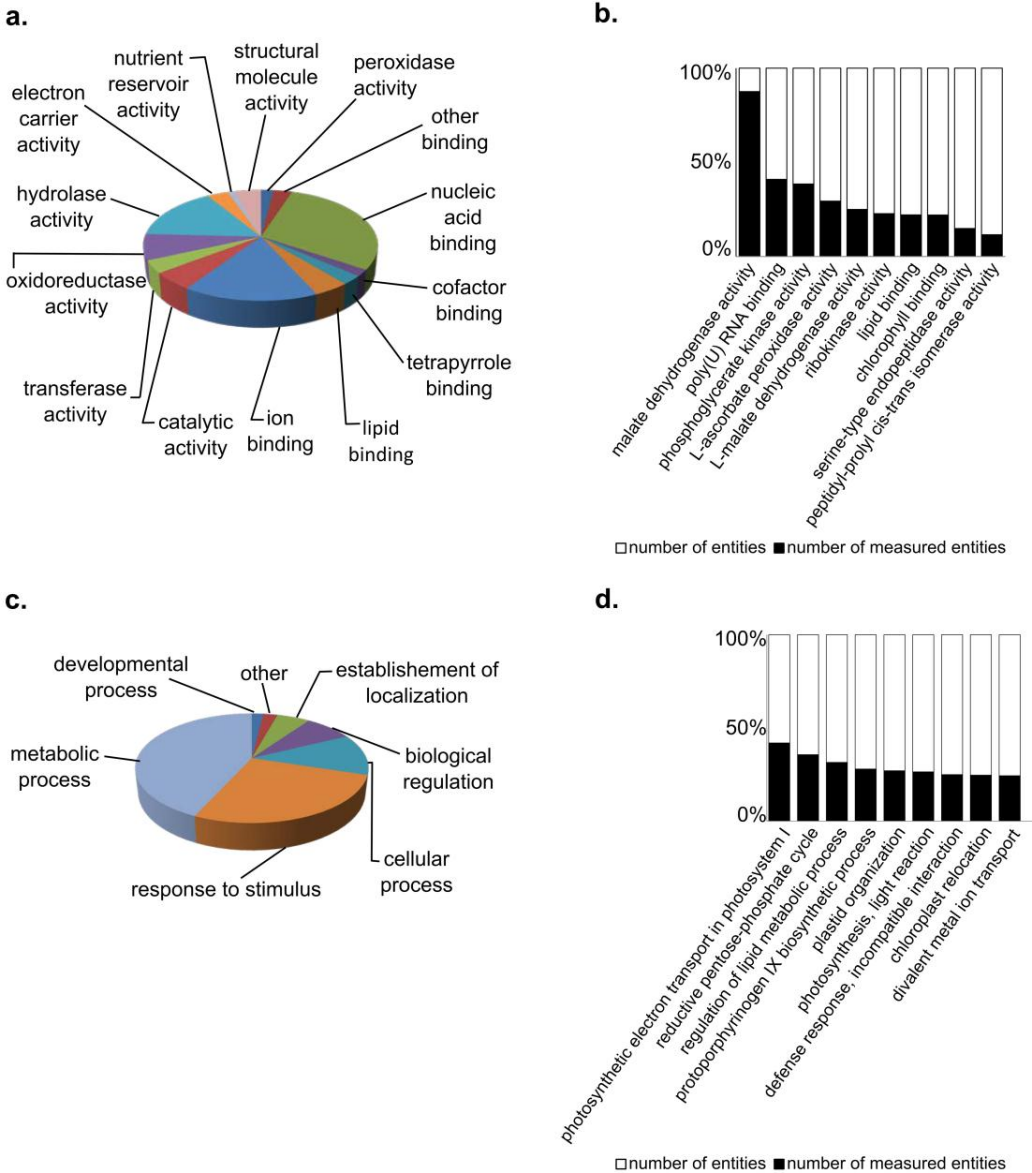
Gene Ontology terms for protein species detected in bean leaves. Gene Ontology (GO) terms were assigned based on a library for *Arabidopsis thaliana.* (**a**) GO terms for molecular function represented as the total number of proteins with each GO term; (**b**) The 10-most enriched molecular functions as determined based on the percent of the biochemical pathway represented (number of entities detected/number of entities in the library × 100%); GO terms were similarly evaluated for (**c**) biological process and (**d**) biological process-enriched pathways.

The 640 detected bean protein species were categorized based on annotated gene ontology (GO) terms (Figure 2, Supplemental Table S3). The results indicated that the detected protein species are involved in many metabolic processes, for example proteins associated with response to stimuli and that contain binding activities. For example, 43 protein species were found to be involved in defense responses to bacterium, 48 protein species were involved in photosynthesis, 81 protein species identified in ATP binding, and 63 protein species involved in the response to salt stress (Supplementary Table S3).

While the use of GO terms is instructive for global characterization of proteomes, our results illustrate the limitation of this approach when working with non-model systems. Specifically, there were eight protein species (sedoheptulose bisphosphatase, fructose-bisphosphate aldolase 2, ATP synthase δ-subunit gene, phosphoglucomutase, ascorbate peroxidase 4, Mog1/PsbP/DUF1795-like photosystem II reaction center PsbP family protein, starch branching enzyme 2.2, and NDH-dependent cyclic electron flow 1) for which GO terms indicated involvement in glucosinolate metabolic process. All eight protein species were also found to be involved in other molecular functions. To our knowledge, glucosinolates have not been detected in common bean*,* and the molecular function of bean proteins may be incorrectly classified due to the reliance on homology to the *Arabidopsis thaliana* GO term library.

**Figure 3:**
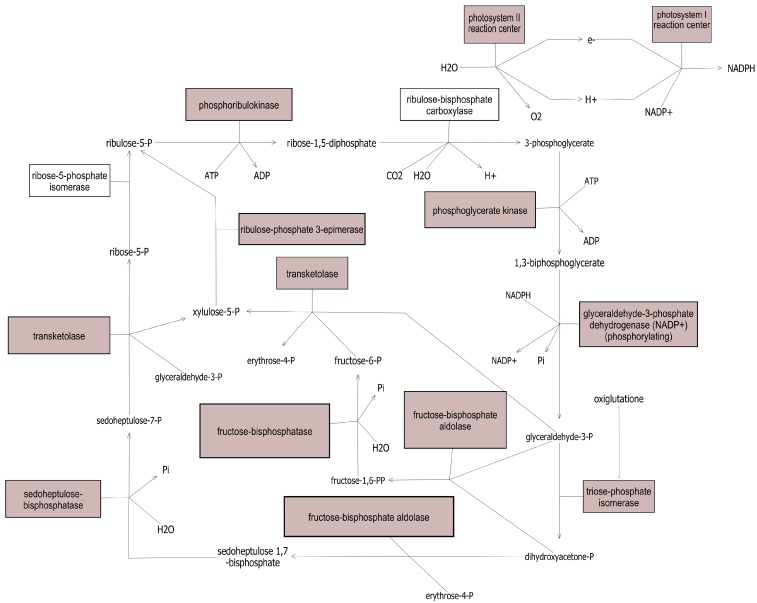
Detected protein species associated with photosynthesis. Proteins detected and identified in this study are shaded in grey.

Gene set enrichment analysis (GSEA) was also performed to identify biochemical pathways highly represented in the protein dataset (Supplementary Table S4). GSEA characterizes categorical bias within the list of protein species to identify shared functions or properties in systems biology [26]. GSEA analysis of the 640 detected protein species identified 74 pathways as enriched (Mann-Whitney-U test, *p* < 0.05). The most enriched pathways included photosynthesis and defense response pathways. For example, 13 of 15 protein species (86.67%) in the photosynthesis pathway were detected (Figure 3). An analysis of total identified protein species revealed 41 protein species involved of hormone signaling and biosynthesis with overlap of shared proteins. For example, 20 detected protein species are listed as involved in both jasmonic acid and salicylic acid pathways. Thus, future studies could utilize this approach to study the crosstalk between jasmonic and salicylic acid pathways by monitoring proteins involved in the defense network.

### 3.2. Quantitative Variation in Proteins between Bean Lines

Label free quantitation by spectral counting and total MS/MS ion current revealed 29 protein species that varied between line A195 and the Sacramento (Student’s *t*-test, *p* ≤ 0.05). (Table 1, Supplementary Table S5). Not surprisingly, this list includes proteins involved in photosynthesis and response to biotic stress. Specifically, five protein species are involved in plant defense and five are involved in photosynthesis (Table 1). For example, Metacaspase 5 is a proteolytic enzyme involved in hydrogen peroxide-induced programmed cell death and was observed to be in higher abundance in Sacramento as compared to A195 [28,29].

**Table 1:** Protein species significantly different in abundance between A195 and Sacramento.

Go Term	Protein Name	Accession Number	*p*-Value	Fold Change *
Photosynthesis	light harvesting complex photosystem II subunit 6	Phvul.006G207300.1|PACid:27166485	0.0031	145.40
Photosynthesis	light-harvesting chlorophyll B-binding protein 3	Phvul.005G085800.1|PACid:27148435	<0.00010	63.33
Photosynthesis	light harvesting complex photosystem II subunit 6	Phvul.002G207500.1|PACid:27170403	<0.00010	53.33
Oxidation-Reduction, Defense	plastid transcriptionally active 16	Phvul.011G061600.1|PACid:27152979	0.019	5.97
ATP synthesis coupled proton transport	ATP synthase δ-subunit gene	Phvul.003G211100.1|PACid:27143051	0.049	2.15
--	uridylyltransferase-related	Phvul.005G181700.1|PACid:27149150	0.0052	1.93
Oxidation-Reduction, Defense	2-cysteine peroxiredoxin B	Phvul.001G194100.1|PACid:27163227	0.038	1.62
RNA Binding	elongation factor Ts family protein	Phvul.004G168100.1|PACid:27157476	0.0095	1.62
Ion Binding	copper ion binding;cobalt ion binding;zinc ion binding	Phvul.002G265700.1|PACid:27169903	0.035	1.44
Oxidation-Reduction, Defense	Oxidoreductase, zinc-binding dehydrogenase family protein	Phvul.003G060300.1|PACid:27143379	0.018	0.83
Oxidation-Reduction	thioredoxin M-type 4	Phvul.005G071200.1|PACid:27149735	0.043	0.77
--	RNA-binding (RRM/RBD/RNP motifs) family protein	Phvul.005G039000.1|PACid:27150205	0.053	0.69
--	CP12 domain-containing protein 2	Phvul.001G212900.1|PACid:27164757	0.0072	0.67
--	protochlorophyllide oxidoreductase A	Phvul.005G083700.1|PACid:27149633	0.048	0.67
Translation	Ribosomal L5P family protein	Phvul.008G059200.1|PACid:27153603	0.022	0.60
Photosynthesis	cold, circadian rhythm, and rna binding 2	Phvul.009G023700.1|PACid:27146551	0.027	0.53
--	FASCICLIN-like arabinogalactan 2	Phvul.008G288800.1|PACid:27153618	0.036	0.51
Defense	metacaspase 5	Phvul.011G180300.1|PACid:27150868	0.015	0.45
Photosynthesis	2Fe-2S ferredoxin-like superfamily protein	Phvul.002G045400.1|PACid:27168785	0.017	0.33
Protein Binding	DnaJ/Hsp4Unknown cysteine-rich domain superfamily protein	Phvul.003G164500.1|PACid:27144762	0.03	0.30
--	Ribosomal protein S19e family protein	Phvul.005G018600.1|PACid:27149117	0.089	0.30
--	FASCICLIN-like arabinogalactan-protein 1 Unknown	Phvul.008G075000.1|PACid:27155369	0.079	0.22
Lipid Metabolic Process	GDSL-like Lipase/Acylhydrolase superfamily protein	Phvul.006G169100.1|PACid:27166530	0.045	0.20
--	FKBP-like peptidyl-prolyl cis-trans isomerase family protein	Phvul.011G051700.1|PACid:27151439	0.01	0.15
Translation	plastid ribosomal protein l11	Phvul.004G090600.1|PACid:27158071	0.0011	0.13
Protein Binding	Embryo-specific protein 3, (ATS3)	Phvul.005G177100.1|PACid:27149941	0.0023	0.03
Response to Stress	Unknown	Phvul.003G074600.1|PACid:27143731	0.0001	0.01
Oxidation-Reduction, Defense	Peroxidase superfamily protein	Phvul.007G082600.1|PACid:27161387	0.00015	0.01
Ion Binding	allantoinase	Phvul.006G186800.1|PACid:27164938	0.0039	0.01

* Calculated as A195/Sac.

Taken together, the variation in leaf protein abundances between A195 and Sacramento support the utility of the shotgun proteomic approach for future studies to investigating pathways associated with photosynthesis, redox, and pathogen resistance.

## 4. Conclusions

The objective of this study was to demonstrate the utility of shotgun proteomics as a molecular tool for the characterization of beans. Here, we report the detection and confident identification of 640 protein species from bean leaf extracts. Among these, 29 protein species were found to be significantly different in abundance (*p* < 0.05) between A195 and Sacramento, two bean lines that vary in their physiological responses to biotic stress*.* Characterization of the identified protein species revealed their involvement in important pathways including oxidation/reduction, photosynthesis, and defense against pathogens.

Mapping of the protein species against the bean genome demonstrates the broad coverage of detected protein species across the genome and the utility of this approach for the identification of protein QTL in the context of pathogen infection. Overall, the results presented here demonstrate the effectiveness of the shotgun proteomics approach for the analysis of plant tissue and the potential for this technique to elucidate proteome changes indicative of plant defense mechanisms. While a shotgun proteomics approach will be biased towards the most abundant proteins, our results illustrate that a sufficient breadth of proteome coverage is achieved to inform on important biological pathways and mechanisms. Importantly, this proteome characterization lays the groundwork for future experiments to focus on host proteome changes in response to pathogen inoculations which could improve our understanding of plant resistance mechanisms.

## Supplementary Materials

Supplementary File 1
